# Transgene Detection by Digital Droplet PCR

**DOI:** 10.1371/journal.pone.0111781

**Published:** 2014-11-06

**Authors:** Dirk A. Moser, Luca Braga, Andrea Raso, Serena Zacchigna, Mauro Giacca, Perikles Simon

**Affiliations:** 1 Faculty of Psychology, Genetic Psychology, Ruhr-University-Bochum, Bochum, Germany; 2 International Centre for Genetic Engineering and Biotechnology (ICGEB), Molecular Medicine, Trieste, Italy; 3 Department of Sports Medicine, Disease Prevention and Rehabilitation, Johannes Gutenberg-University Mainz, Mainz, Germany; H. Lee Moffitt Cancer Center & Research Institute, United States of America

## Abstract

Somatic gene therapy is a promising tool for the treatment of severe diseases. Because of its abuse potential for performance enhancement in sports, the World Anti-Doping Agency (WADA) included the term ‘gene doping’ in the official list of banned substances and methods in 2004. Several nested PCR or qPCR-based strategies have been proposed that aim at detecting long-term presence of transgene in blood, but these strategies are hampered by technical limitations. We developed a digital droplet PCR (ddPCR) protocol for Insulin-Like Growth Factor 1 (*IGF1*) detection and demonstrated its applicability monitoring 6 mice injected into skeletal muscle with AAV9-*IGF1* elements and 2 controls over a 33-day period. A duplex ddPCR protocol for simultaneous detection of Insulin-Like Growth Factor 1 (*IGF1*) and Erythropoietin (*EPO*) transgenic elements was created. A new DNA extraction procedure with target-orientated usage of restriction enzymes including on-column DNA-digestion was established. *In vivo* data revealed that *IGF1* transgenic elements could be reliably detected for a 33-day period in DNA extracted from whole blood. *In vitro* data indicated feasibility of *IGF1* and *EPO* detection by duplex ddPCR with high reliability and sensitivity. On-column DNA-digestion allowed for significantly improved target detection in downstream PCR-based approaches. As ddPCR provides absolute quantification, it ensures excellent day-to-day reproducibility. Therefore, we expect this technique to be used in diagnosing and monitoring of viral and bacterial infection, in detecting mutated DNA sequences as well as profiling for the presence of foreign genetic material in elite athletes in the future.

## Introduction

Somatic gene therapy represents a promising tool to treat inherited or acquired diseases by transferring genetic material in order to compensate for defective genes, to produce a therapeutic substance, or to specifically trigger the immune system [1]. Despite its potential to treat life-threatening diseases, this technique might also be abused to improve physical performance [2]. Animal studies demonstrated successful viral transfer of potential performance-enhancing genes such as Insulin-Like Growth Factor 1 (*IGF1*; [3], [4]) and Erythropoietin (*EPO*; [5]) but severe adverse events were also reported [6]–[9]. This has raised concerns about the illicit use of gene transfer technologies in elite sports (i.e. gene doping), and demonstrated the necessity to prohibit the use of gene transfer aimed at enhancing performance (WADA 2004), with the consequent need to monitor for the presence of transgenic DNA in routine gene doping tests.

The main obstacle for gene-doping detection is that the athlete's body would be enabled to produce doping substances that, in most cases, would be indistinguishable from endogenous proteins [10], [11]. Therefore, currently proposed direct detection methods rely on specific sequence characteristics in transgenic DNA constructs that allow unbiased discriminability between genomic and exogenous DNA. Since skeletal muscle (the most likely target tissue for gene doping applications) would be difficult to harvest for routine doping testing, several studies explored the possibility to detect minute amounts of transgenic DNA in the blood after somatic (intramuscular) gene transfer.

In these studies different PCR-based approaches, such as nested PCR [12]–[14] and TaqMan qPCR [8], [15]–[17], were applied in order to detect minute amounts of transgenic DNA in blood. These methods selectively detect and discriminate the intron-less transgene from genomic DNA by PCR. Thus, detection of transgenic DNA in whole blood samples could provide a suitable means to support conviction of unscrupulous athletes. However, current PCR-based detection approaches are either highly sensitive but require a nested PCR procedure with a laborious workflow [12]–[14], or they display weaknesses to sensitively detect minute amounts of the transgene in a single round qPCR, [8], [15], [17], [18]. In addition, qPCR relies on external standard curves, which further complicates inter-lab comparability of results. It is also important to point out that DNA amplification efficiency is highly dependent on template structure in the way that circular DNA amplifies poorly compared to linear DNA [19], [20]. To date, all aforementioned methods use undigested DNA as a template which can result in poor amplification of circular DNA structures and consequently in failure to detect the specific transgenic sequence in a huge background of endogenous DNA. As recombinant DNA is integrated into the host genome or persists as episomal circular supercoiled DNA, predigestion of DNA should improve PCR sensitivity and increase the likelihood of transgene detection.

Digital droplet PCR (ddPCR) is a new method that enables the absolute measure of target DNA. Its principle is based on the portioning of PCR mixture into thousands of droplets per reaction [21]. As ddPCR can also handle huge amounts of background DNA in the reaction, it represents a convenient method to find the transgenic “needle in a haystack”. Additional benefit of ddPCR over qPCR also includes absolute quantification, which does not rely on external standard curve, leading to excellent day-to-day reproducibility. This is why ddPCR is likely to become a favourite tool for accurate routine analysis, especially when performed at multi-site laboratories.

Here we describe a new digital droplet PCR assay for transgene *IGF1* detection after intramuscular AAV9 gene transfer. DNA was purified from whole blood of 6 intramuscularly AAV9-*IGF1* transduced mice and 2 uninjected control animals, which were monitored for 33 days for the detectability of transgene elements by ddPCR.

We also developed a duplex ddPCR protocol for *IGF1* and *EPO* transgene elements to simultaneously detect two candidate genes for gene doping in a single assay. We further established a method to test for ddPCR efficiency by the addition of an internal control standard (ICS) at a defined copy number to the reactions, a method already described for qPCR elsewhere [17]. This ICS differs only from the transgene in its probe binding site and can be detected in parallel to the transgene, which makes monitoring of ddPCR efficiency feasible in each reaction.

To minimize sample handling and to optimize for transgene detectability, we also developed a new DNA extraction protocol, which includes on-column DNA restriction enzyme digestion and allows the elution of fragmented high quality DNA as optimal target for ddPCR. This procedure leads to further improved sensitivity of the assay and might also find application for assays where preferential amplification of target sequences is warranted.

Thus we aim at presenting a straightforward and highly sensitive approach for DNA detection, which could lead to the next generation of transgene detection.

## Methods

### DNA extraction, digestion and purification

Human genomic DNA (hgDNA) for spike-in experiments was extracted at large scale using the salting-out procedure as described by Miller et al. [22]. PCR standards of the *IGF1* and *EPO* coding-sequence were generated using the primers and resulting amplicon lengths as indicated in Table 1. To generate circular standards, purified PCR products were cloned into the PCRII-TOPO vector according to the manufacturer's recommendations. All standards were Sanger-sequenced to check for correctness of the sequences. DNA was spiked with defined copy numbers of freshly prepared ddPCR quantified *IGF1-* or *EPO* standard, and digested using restriction enzymes DdeI and RsaI (2 units/µg hgDNA) in NEB buffer 2 for 1 h at 37°C followed by 20 minutes heat inactivation at 65°C.

**Table 1:** Primer and Probe sequences.

Gene	Primer and Probe sets in 5→3 orientation [concentration]	Amplicon size
Erythropoietin (*EPO*); NM_000799	Fw: TGAATGAGAATATCACTGTCCCAGAC [900 nM] Rev: CTTCCGACAGCAGGGCC (900 nM); P: [Hex]AAG[+A]GG[+A]TG[+G]AG[+G]TCGG[BHQ1] [250 nM]; Sigma	114 bp
	Coding sequence primers: Fw: ATGggggtgcacgaatgt; Rv: TCAtctgtcccctgtcctg	582 bp
Insulin-like growth factor 1; (IGF1) NM_000618.3	Fw: GCTGGTGGATGCTCTTCAGTT [900 nM] Rev: TCCGACTGCTGGAGCCATAC [900 nM] P:[FAM]CTT[+T]TA[+T]TT[+C]AA[+C]AA[+G]CC[+C]AC[BHQ1] [250 nM]; Sigma	83 bp
	Coding sequence primers: Fw: ATGggaaaaatcagcagtcttc; Rv: CTAcatcctgtagttcttgtttcctg	462 bp
**I**nternal **C**ontrol **S**tandard (ICS)	Fw: GCTGGTGGATGCTCTTCAGTT [900 nM] Rev: TCCGACTGCTGGAGCCATAC [900 nM] P: [VIC]TGCTCCAGAGAAGAAACCAC[MGB-NFQ] [250 nM]; Life Technologies	82 bp

Forward (Fw), reverse (Rev) primer-, and probe (P) sequences inclusive corresponding amplicon lengths. Start and stop codons in the coding sequence primers are capitalized.

### Production, purification, and characterization of rAAV vectors

The human hepatic *IGF1-IA* (ref. seq. NM_000618.3) was PCR amplified and cloned into the pZac recombinant AAV expression vector, generating the pAAV-*IGF1* vector. Viral particles were produced by the AAV Vector Unit at ICGEB Trieste (http://www.icgeb.org/avu-core-facility.html). Methods for production and purification were previously described [23]. AAV9 titers were in the range of 1×10^13^ genome copies per milliliter.

### AAV9-*IGF1* injection into the skeletal muscle

Animal care and treatments were conducted in conformity with institutional guidelines in compliance with national and international laws and policies, upon approval by the ICGEB Ethical Committee and by the Italian Minister of Health (EEC Council Directive 86/609, OJL 358, December 12, 1987). Animals were provided with housing in an enriched environment, with at least some freedom of movement, food, water and daily care and cleaning. Experiments were performed under general or local anesthesia, and with constant use of analgesics. At the end of any experiment, competent authorized persons decided the proper time and most appropriate humane method for animal sacrifice. All experiments were performed in male CD1 mice, 4–6 weeks of age. As a model of gene doping, tibialis anterior and gastrocnemius muscles were injected with 50 µl of either PBS or a viral suspension containing 10^11^ viral particles of AAV9-*IGF1*, and harvested after the indicated periods of time.

### DNA extraction and digestion from mouse blood

DNA was extracted from 100 µl mouse blood using the Qiagen DNA microkit, which enables the elution of DNA in variable volumes between 20–100 µl. DNA was eluted in a volume of 25 µl and digested with 5 U DdeI and RsaI in a final volume of 30 µl at 37°C for 1 h followed by heat inactivation at 65°C for 20 minutes.

Restriction enzymes DdeI and RsaI were chosen according to the following criteria (see also Table 2):

**Table 2:** Number of restriction sites for *Dde*I and *Rsa*I in the human/mouse genome and at the *IGF1* and *EPO* gene locus.

	*Dde*I (CTNAG)	*Rsa*I (GTAC)
Approximate number of cutting sites per Mbase in the human genome	**4,844.1** *	**1,764.1** *
average fragment length	**206** *	**567** *
Approximate number of cutting sites per Mbase in the mouse genome	**5,291.6** *	**2,122.4** *
average fragment length	**189** *	**471** *
***IGF1***		
*IGF1* gene locus (human); chr12: 102789645–102874378	**432**	**147**
*Igf1* gene locus (mouse); chr10: 87859056–87937047	**430**	**129**
*Human IGF1 gene locus* (56035 bp between primers); chr12: 102813436–102869470	**293**	**100**
*IGF1* mRNA (7321 nucleotides)	**28**	**12**
*IGF1* cds (462 nucleotides)	**3**	**1**
*IGF1* amplicon (83 nukleotides)	**0**	**0**
***EPO***		
*EPO* gene locus (human); chr7: 100318423–100321323	**19**	**4**
*Human EPO gene locus* (729 bp between primers); chr7: 100319610-100320338	**5**	**0**
*EPO* mRNA (1340 nucleotides)	**10**	**2**
*EPO* cds (582 nucleotides)	**1**	**2**
*EPO* amplicon (114 nucleotides)	**0**	**0**

* Information taken from http://tools.neb.com.

- they do not cut the coding sequence between the primers
- they cut the *IGF1* and *EPO* coding sequence 5′ and/or 3′ of the PCR-amplicon which linearizes potential circular viral or plasmid constructs irrespective of any knowledge of the vector sequence
- they frequently cut the *IGF1* and *EPO* intronic region to prevent background amplification of their genomic locus during PCR
- in order to exclude the effects of endogenous CpG methylation on restriction enzyme activity, no CpG sites are allowed to be present in the restriction enzyme recognition sites

### qPCR

*IGF1*-specific primers and probe were chosen to target all *IGF1* mRNA isoforms including mechano-growth factor (*MGF*) using UCSC Genome Browser (http://genome.ucsc.edu/) and Primer 3 [24]. Then, *IGF1* qPCR was optimized and tested for the limit of detection/limit of quantification (LOD/LOQ) as described by Burns et al. [25] using standard calibrators in a background of 500 ng hgDNA. The LOD was estimated by 2-fold serial dilutions between ∼2000 and 1 calibrator copies per reaction (See Figure S1). We defined the LOD as the lowest copy number that gives a detectable PCR amplification product at least 95% of the time. The LOQ was defined as the lowest concentration that could be quantified with >80% accuracy, and LOD was defined as the minimum copy number for which all replicates of the same dilution could be successfully detected. qPCR mixture contained 10 µl SsoFast probes supermix (Bio-Rad) and primers and probes as indicated in Table 1. Two-step PCR protocol using CFX384 (Bio-Rad) started with 2 min at 98°C followed by 45 cycles at 95°C melting and 30 sec annealing/extension at 64°C.

To test for putative template effects on amplification efficiencies, two different sized plasmids containing the 462 bp *IGF1* coding sequence (pAAV9-*IGF1*-5237 bp; Topo PCR 2.1-*IGF1*-4339 bp) and a PCR-generated *IGF1* standard were subjected to qPCR with or without DdeI and RsaI double-digestion (See Figure S2).

### Nested-qPCR

As a positive control, nested qPCR was performed to test for the presence of AAV9-*IGF1* elements in DNA isolated from mouse blood. The first round PCR was done using 4 µl of DNA using primers and conditions as described earlier [13]. PCR-products were 1∶50 diluted in water and two microliter subjected to second round amplification (in triplicates) using the primers and conditions as described above. After qPCR, nested-qPCR products were analysed on a 2.5% agarose gel.

### ddPCR

Each PCR reaction consisted of a 20 µL solution containing 10 µL ddPCR supermix for probes (Bio-Rad), 900 nM primers, 250 nM probe and 4 µl template DNA. Droplets (∼20.000/reaction) were generated on the Bio-Rad QX-100 following the manufacturer's instructions. Samples were transferred on a 96 well-plate and thermal cycled to the endpoint (T100 Thermal Cycler; Bio-Rad) using a standard protocol; initial denaturation at 95°C for 10 min, followed by 40 cycles of melting at 95°C for 30 seconds and annealing/elongation at 61°C for 1 minute, before droplet stabilisation by 10 min incubation at 98°C. After cycling, the 96 well-plate was immediately transferred on a QX100 Droplet Reader (Bio-Rad) where flow cytometric analysis determined the fraction of PCR-positive droplets vs. the number of PCR-negative droplets in the original sample. Data were analysed using Poisson statistics to determine the target DNA template concentration in the original sample. Optimal annealing temperatures were assayed carrying out gradient PCR for all primers and their specific targets (See Figure S3). Subsequently, we tested for potential inhibitory effects of restriction buffer on ddPCR and observed inhibitory effects only in the cases when more than 5 µl of DNA restriction solution (2.5 mM NaCl; 500 nM Tris-HCl; 500 nM MgCl_2_; 50 nM DTT) were subjected to 20 µl ddPCR (Figure S4 a). Increasing amounts (up to 1500 ng) of genomic DNA in the background of the reaction were also assayed and did not show any inhibitory effects on ddPCR (Figure S4 b). Consequently, all ddPCRs were performed using a final volume of 4 µl template DNA (47 ng–1500 ng), which avoided potential salt and DNA inhibitory effects.

### ddPCR detection of *IGF1* transgene from mouse blood and human spike-in experiments

For transgene detection, human/mouse genomic DNA was extracted as described above and 4 µl subjected to ddPCR using the primers and probes at concentrations as indicated in Table 1.

### *IGF 1* and *EPO* ddPCR-duplex assay

We also established a duplex protocol for parallel *IGF1* and *EPO* ddPCR transgene detection. *EPO*-specific primers and probe were used as described elsewhere [17]. Serially diluted standards (∼5000 copies were 1∶5, 1∶2, 1∶5, 1∶2, 1∶5 diluted) were assayed using ddPCR chemistry containing 900 nM *IGF1* and *EPO* forward and reverse primers, supplemented with differentially labelled *IGF1* and *EPO*-specific LNA TaqMan-probes at 250 nM final concentration (see Table 1). *EPO*-specific primers and probe were used as described elsewhere [17]. Samples were assayed in a background of 500 ng DdeI and RsaI fragmented human genomic DNA containing serial dilutions of either *IGF1* or *EPO* standards only; both standards at decreasing levels and also containing *IGF1* at decreasing and *EPO* at increasing amounts. Digital droplet PCR was performed under standard conditions as described above.

### ICS (internal control standard)

To check for uniform PCR efficiency all reactions were spiked with an internal standard (similar to the internal threshold control (ITC) as described for qPCR [17]). This artificial standard (purchased and synthesized by MWG Eurofins) was designed to have the identical 5′ and 3′ sequence compared to the *IGF1* amplicon, but with the probe binding site replaced by a sequence taken from the ancestral organism Cyanobacterium stanieri (NC_019778.1). This 20 nucleotide sequence was blasted against the human genome to confirm that there was no presence of either identical or similar sequence, which could lead to false positive detection. A VIC-labelled TaqMan probe (Life Technologies) was designed to target this sequence in a duplex PCR when *IGF1* transgenic elements are also detected.

Subsequently, *IGF1* standard was assayed using ddPCR chemistry supplemented with an internal-control standard at defined concentration (100 copies/reaction).

### DNA extraction by on-column digestion

Duplicates of whole blood samples of 5 human donors (100 µl) were spiked with the same amount of circular standard DNA. Using the Qiagen DNA microkit, DNA was extracted and was finally processed using 3 different procedures as follows:

- DNA was eluted conventionally with 30 µl H_2_O.
- Water-eluted DNA was DdeI and RsaI digested (5 U each) for 1 h at 37°C.
- DNA was on-column digested for 1 h at 37°C with 30 µl of a solution containing 10 U DdeI and RsaI in 1 x buffer 2.

All samples were adjusted to the same salt concentration and dilution, heat inactivated for 20 min at 65°C, Nano-dropped, agarose-gel visualized and subsequently ddPCR quantified under the conditions as described above.

## Results

As a preliminary step towards ddPCR, qPCR experiments were performed to identify those primers and probes, which led to best PCR efficiencies, and which did not produce artefacts such as excessive by-products or false positive signals. Accordingly, primers and probes as indicated in Table 1 were used.

### qPCR

We designed a new assay for *IGF1* transgene detection with primers that resulted in an 83 bp amplicon, in which the exon2/3 boundary was targeted by a 6-FAM-labelled LNA-probe. As illustrated in Figure S1, *IGF1* qPCR efficiency was 96.7% with a linearity of r^2^ = 0.98-. The LOQ was defined as the lowest concentration that could be quantified with >80% accuracy, and set to 16 copies per reaction (See Figure S1); LOD was found to be 4 copies. We then tested for differential PCR efficiency dependent on DNA structure. We assayed serial dilutions (ranging from 10^6^–10 copies) from 2 vectors carrying the *IGF1* coding sequence (pAAV9-*IGF1*-5237 bp and TOPO-*IGF1-*4397 bp) compared to linear PCR product. Improved amplification for the linear PCR standard (Ct difference of more than 3), compared to circular, supercoiled vectors was observed. Subsequently, we tested digested plasmid vs. undigested plasmid compared to linear PCR-standard and were able to amplify the digested plasmid with efficiencies close to the linear PCR-standard (See Figure S2).

### ddPCR optimization

To determine best target-specific annealing temperatures, all primers used in ddPCR were initially tested by gradient ddPCR. As indicated in Figure S3, all primers worked well between 66°C–60°C with highest amplitude and best sensitivity at ∼61°C. Subsequently, all ddPCR assays were conducted at 61°C annealing/extension temperature. Furthermore, we verified that genomic DNA, up to 1500 ng, in the ddPCR reaction did not affect results (See Figure S4 a), and that 1 x restriction solution, less than 5 µl, added to ddPCR did not inhibit the reaction (See Figure S4 b). Consequently, 4 µl of restriction solution were routinely used for transgene detection.

### ddPCR for AAV9-*IGF1* transgene detection in mice

**Figure 1:**
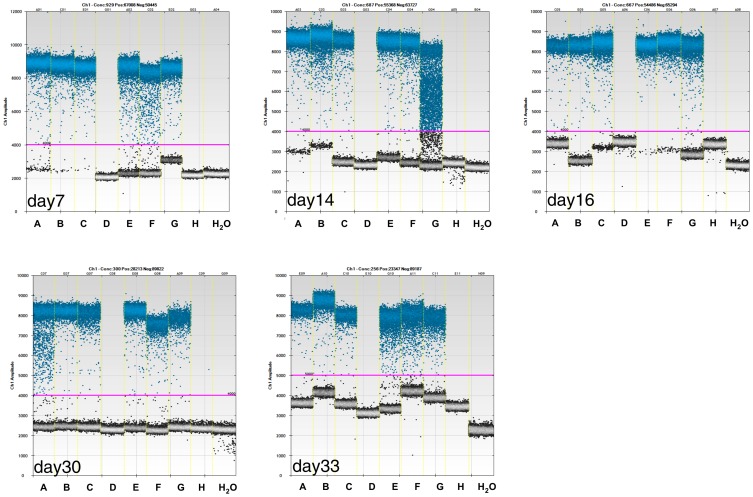
*IGF1* transgene detection by ddPCR. Eight mice, 6 AAV9-IGF1 muscle transduced (A, B, C, E, F, G) and two controls (D and H) were screened for 33 days by ddPCR for *IGF1* transgene detectability.

Our aim was to detect minute amounts of *IGF1* transgenic DNA using ddPCR, a method so far never described for gene-doping detection. From 100 µl whole blood, DNA was extracted with 25 µl H_2_O and digested in NEB buffer 2 using the restriction enzymes DdeI and RsaI, in a final volume of 30 µl. Four µl of digested DNA were subjected to PCR in 3 independent trials, and *IGF1* was detected as illustrated in Figure 1. Results provided specific indication of *IGF1*-transgene detection in all transduced animals over a 33 day-period. As displayed in Table S1, copy numbers were in the range of 3200–164800 copies/reaction (which indicates 240–12263 viral elements/µl of whole blood) also indicating excellent ddPCR reproducibility with a tendency to lower values, due to additional thawing and freezing cycles. Control animals remained negative with one single false positive event (Fig 1-day30 and Figure S5). However, this false positive event could be clearly discriminated from true positives by manual re-adjustment of the threshold to a value that defined the lower limit of the positive control, a best practice for ddPCR, as also discussed earlier elsewhere [26].

Digital droplet PCR results were confirmed by nested-qPCR and by gel-electrophoresis of the corresponding PCR-products. PCR products showed by-products of similar size as the expected *IGF1* PCR-products, however, these by products were not detected by the *IGF1*-specific probe during qPCR (data not shown).

### Spike-in duplex assay for simultaneous *IGF1* and *EPO* detection at low copy numbers

To ameliorate the use of the limited amount of DNA extracted from blood and to optimize the flow capacity of a potential routine transgene detection setting, we also developed a new duplex ddPCR assay for simultaneous *IGF1* and *EPO* detection.

**Figure 2:**
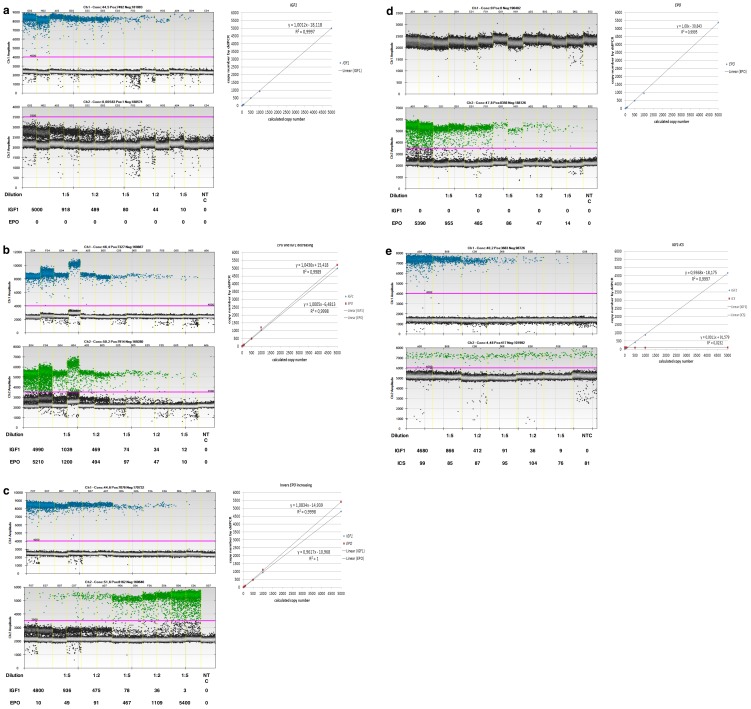
Duplex ddPCR for *IGF1* and *EPO* transgene elements. 500 ng human gDNA were spiked with serial dilutions (1∶5; 1∶2; 1∶5; 1∶2 and 1∶5) of *IGF1*-standard (A), decreasing amounts of *IGF1* and *EPO*-standard (B), inverse amounts of *IGF1* (decreasing) and *EPO* (increasing) (C), *EPO* only (D) and *IGF1* multiplexed with ICS at ∼100 copies/reaction (E). Displayed are amplitudes, copy numbers per 20 µl reaction and linearity of the signals.

Serially diluted *IGF1* and *EPO* standards were spiked into 500 ng human genomic DNA and analysed by ddPCR after DdeI and RsaI digestion. Digital droplet PCR was performed using a mixture containing *IGF1* and *EPO* primers and probes. This test should reveal sensitivity and linearity of the assay for single transgene detection and also in a duplex approach at low copy numbers (range from ∼5000–10 copies/reaction). As indicated in Figure 2 (A–E), both transgenes were detected showing linearity values from 0.9997 to 1 for *IGF1* and 0.9995 to 0.9998 for *EPO* under the conditions tested. Sensitivity and linearity of the duplex assay did not differ from those obtained by the single gene assay.

*IGF1* standard was also assayed in the presence of an internal control standard at ∼100 copies/reaction. As indicated in Figure 2E, ICS was clearly detected in all reactions, and could be used as a reporter for PCR efficiency in each run and for all reactions. This minimizes probability of false negatives due to potential PCR inhibitors being present in some samples.

### DNA extraction by on-column digestion

We also optimized a protocol of on-column digestion prior to DNA fragmentation. This procedure led to fragmented high quality DNA, with final yields that were on average 3 times higher compared to conventionally eluted DNA as revealed by NanoDrop 1000, agarose-gel analysis (See Figure S6).

**Figure 3:**
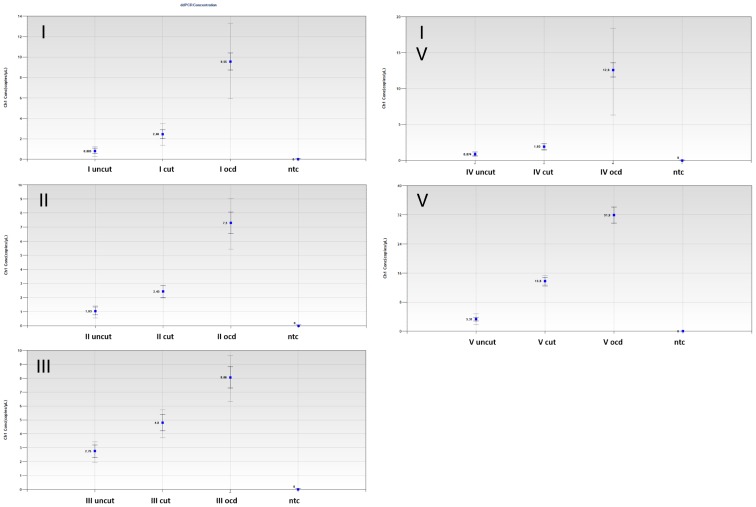
DdPCR results following 3 different DNA extraction procedures. Blood of 5 donors was spiked with the same amount of circular plasmid containing the *IGF1* coding sequence. DNA was extracted using 3 different procedures: uncut, DdeI and RsaI cut, and on-column DdeI and RsaI digested (ocd). Results are displayed as copies detected per µl for 5 subjects (I–V for uncut; conventionally digested (cut), on-column digested DNA (ocd), and no-template control (ntc). Results derive from two independent experiments performed in duplicates.

In addition, testing DNA extracted from whole blood (a- conventionally extracted; b- conventionally extracted followed by DNA digestion; c- on-column digested) that was spiked with the same amount of plasmid standard by ddPCR revealed more sensitive detection for digested DNA, and most sensitive detection for samples subjected to on-column digestion. As indicated in Figure 3, we could achieve 1.9–2.8 fold increased ddPCR sensitivity comparing digested DNA to undigested DNA. When ddPCR was performed from the same blood after on-column digestion, ddPCR performance was further increased 2.9–19 fold compared to DNA eluted using water (Figure 3).

## Discussion

Digital droplet PCR represents a new technical approach for applications where conventional PCR meets its technical limits, such as rare event detection in the presence of high amounts of genomic DNA. Compared to conventional qPCR, quantification by ddPCR does not rely on external standard curves and is more tolerant to inhibitors and variation in amplification efficiencies. Thus, ddPCR is well suited for the identification of transgenic elements with the objective of gene-doping detection.

Here we present a new ddPCR method for *IGF1* and *EPO* transgene detection with high sensitivity and reliability, in combination with an effective technique to isolate high amounts of fragmented DNA with minimal effort. The aim of this study was to test and optimize detection of transgenic elements by the use of ddPCR. Accordingly, we initially tested *IGF1*-specific primers and probes by qPCR for optimal primer annealing, amplicon generation and specific probe detection. Quantitative PCR-experiments revealed a LOQ of 16 and a LOD of 4, which indicated highly efficient PCR conditions (See Figure S1). This qPCR-assay was subsequently transferred to ddPCR and re-optimized for best annealing temperature (See Figure S3) and controlled for either salt or DNA inhibition using (See Figure S4) ddPCR specific chemistry.

Eight mice, of which 6 were transduced with AAV9-*IGF1* and 2 controls, were then monitored over a 33-day period for the detectability of transgenic elements extracted from whole blood. Strong signals could be detected for all AAV9-*IGF1* transduced mice at all points in time, with a tendency of signal reduction over time (Figure 1 and Table S1). We also created a ddPCR duplex assay aimed at the simultaneous detection of *IGF1* and *EPO* detection. As indicated in Figure 2 (A–E), both transgenes were sensitively detected showing linearity from 0.9997 to 1 for *IGF1* and 0.9995 to 0.9998 for *EPO* with copy numbers from 5400 to 3. These data are in accordance with published data describing linearity close to 1 with a limit of detection of 5 when performing duplex ddPCR assays for other genes [16].

The integration of an artificially generated internal control standard into ddPCR enabled us to control for PCR efficiency in each well. As indicated in Figure 2E, the documentation of ddPCR efficiency for each reaction represents a valuable tool for routine analyses as it allows for identification of false negatives, potentially originating from PCR inhibitors being present in some reactions.

On the genomic level, *IGF1* primers target exonic elements separated by a ∼56 kb sequence (56035 bp), containing 293 DdeI and 138 RsaI cutting sites. Primers and probe for EPO detection were used as described by others [17] and could generate a genomic fragment of 726 bp which is cut 5 times by DdeI during digestion. DdeI and RsaI restriction enzyme digestion lead to fragmentation of the respective gene loci and inhibition of primer extension after digestion. This, and the design of *IGF1* and *EPO* specific probes which specifically target exon/exon boundaries, makes detection of false positive signals generated from *IGF1* and *EPO* genomic loci unlikely. Additionally, our data demonstrate that amplification of linearized DNA has superior PCR efficiency over circular DNA elements (Figure 3 and Figure S2), which further supports the use of restriction enzyme digestion of DNA prior to PCR. Our optimized DNA-extraction procedure includes an on column-digest at the final step of DNA extraction. This involves minimal handling of samples and results in much higher DNA yield as compared to conventional DNA extraction procedure (Figure 3 and Figure S6). This may be explained by improved elution of fragmented DNA from the column as compared to undigested high molecular DNA stretches. Comparing eluates of blood samples that were spiked with the same amount of circular *IGF1* standard after conventional DNA purification with, and without restriction to on-column digested DNA by ddPCR, we found the latter to be the most efficient substrate for transgene detection. Accordingly, we recommend use of on-column digestion for all applications where minute amounts of DNA need to be detected, as it increases DNA yield and, thus, the probability of target elution. As displayed in Figure 3, linearization of circular target DNA improved (dd-) PCR detection efficiency, which was further enhanced by on-column digestion due to significantly improved DNA elution from the column.

In analogy to trials which aim to isolate low levels of a mutant sequence in a high background of DNA, such as ARMS-PCR (Amplification Refractory Mutation System, or allele-specific PCR; ASP [27]), we believe on-column digestion using carefully chosen enzymes may help to increase detection of specific DNA elements by PCR in the future.

We did not, however, observe a consistent Limit of Blank (LOB) for *IGF1* and *EPO* analysis, as recently described for the detection of mutated *KRAS* elements [28]. This difference might be attributed to different PCR systems, or bigger differences between wild type and transgene detection than present in mutational detection. Occasional false positives could be explained by DNA carry-over due to sample handling as previously described for other high sensitive detection methods [13].

If we compare our ddPCR assay to other transgene detection approaches, such as qPCR or agarose-gel based nested PCR, we observe similar sensitivities, as all methods can identify less than five copies per PCR. However, ddPCR offers to advantages to be independent of an artificial standard curve, with all its confounding variables, and to minimise DNA carry-over by strict reduction of DNA handling steps.

Testing for practicability and cost value of ddPCR, Morisset and co-workers [29] describe similar or improved throughput and cost-effectiveness for ddPCR when compared to qPCR. Additionally, increased usage of ddPCR can be expected to lead to further cost reduction in the future. Guidelines for standardization purposes and creation of Good Laboratory Practice (GLP) procedures for ddPCR have already been described [26] and were applied in this study.

Implementation of ddPCR for transgene detection is further encouraged by data recently presented by Strain and co-workers [30], who showed 5–20 fold improved precision comparing ddPCR to qPCR for the detection of total DNA originating from the human immunodeficiency virus (HIV) and its episomal 2-LTR (long terminal repeat) circles.

Hence, we present a new approach for transgene detection at high and minute copy amounts, including a new DNA extraction and digestion method, which delivers high quality, concentrated and fragmented DNA as an appropriate target for ddPCR.

Digital PCR as presented here, including appropriate restriction enzyme digestion of genomic DNA, represents a promising approach for the detection of minimal DNA fractions. Digital PCR will therefore most probably find implementation to monitor gene-therapy trials, and the possible abuse as gene doping, but also to screen for and monitor viral and bacterial infection, and to analyse food and feed products for xenogeneic components.

## Supporting Information

Figure S1 **Standard curve, amplification plot and calculation for experimental estimation of IGF1 LOD and LOQ.**
(DOCX)

Figure S2 **PCR efficiency for undigested vs. digested IGF1 standards - Linearization of circular DNA leads to improved PCR efficiency and better detection at low copy numbers.**
(DOCX)

Figure S3 **Temperature gradient for ddPCR detection of IGF1, EPO and ICS.**
(DOCX)

Figure S4 **ddPCR efficiency under various conditions.**
(DOCX)

Figure S5 **Qualitative assessment of ddPCR results.**
(DOCX)

Figure S6 **Concentration, purity and integrity of DNA comparing three different DNA extraction procedures.**
(DOCX)

Table S1 **IGF1 transgene copy numbers as detected by ddPCR for 6 AAV9-IGF1 transduced mice and 2 controls at 5 different days.**
(DOCX)
