# Supplementary material for: Transgene Detection by Digital Droplet PCR
Source: PLoS One. 2014 Nov 6;9(11):e111781. doi: 10.1371/journal.pone.0111781 (PMC4222945; doi:10.1371/journal.pone.0111781)
Supplement: Figure S1 — Standard curve, amplification plot and calculation for experimental estimation of IGF1 LOD and LOQ. (DOCX) [file pone.0111781.s001.docx]

**Supplemental Data Figure 1:**


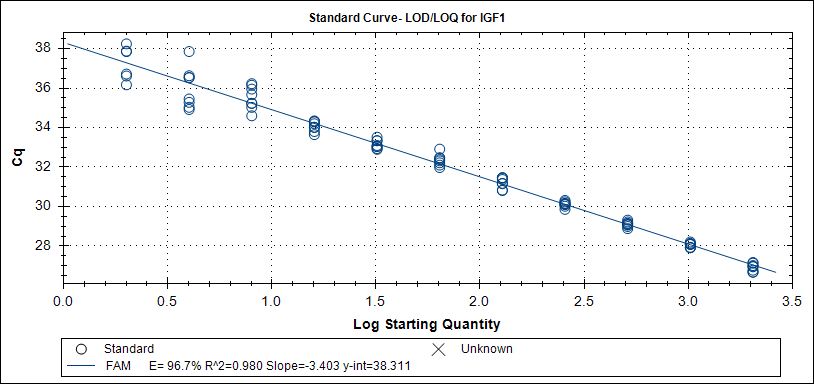

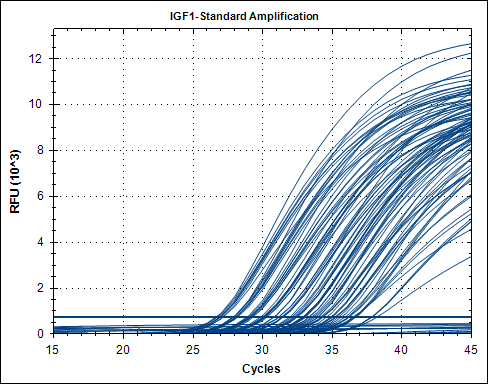

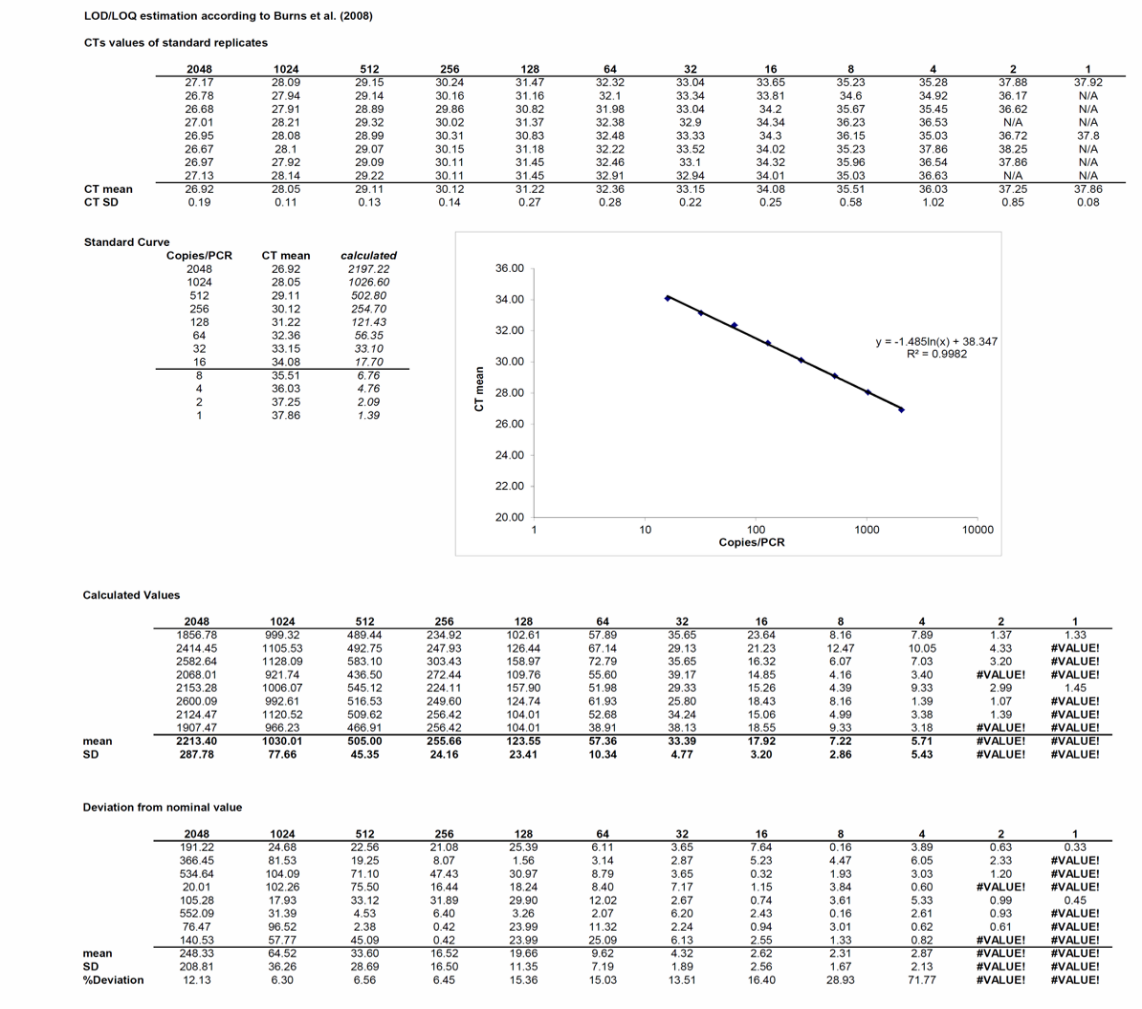


**Supplemental Data Figure 1:** Standard curve, amplification plot and calculation for experimental estimation of *IGF1* LOD and LOQ.

*IGF1* qPCR was optimized and tested for the limit of detection/limit of quantification (LOD/LOQ) as described by Burns et al. [25] using standard calibrators (n=8) in a background of 500ng hgDNA. Freshly quantified standard was serial 1:2 diluted from 2048 copies down to 1 copy/reaction. LOQ for *IGF1* was determined as 16 copies with a limit of detection of 4 copies.

We defined the LOD as the lowest copy number that gives a detectable PCR amplification product at least 95% of the time. The LOQ was defined as the lowest concentration that could be quantified with >80% accuracy, and LOD was defined as the minimum copy number for which all replicates of the same dilution could be successfully detected. qPCR mixture contained 10µl SsoFast probes supermix (Bio-Rad) and primers and probes as indicated in Table 1. Two-step PCR protocol using CFX384 (Bio-Rad) started with 2 min at 98°C followed by 45 cycles at 95°C melting and 30sec annealing/extension at 64°C. We designed a new assay for *IGF1* transgene detection with primers that resulted in an 83bp amplicon, in which the exon2/3 boundary was targeted by a -FAM™-labelled LNA-probe. As illustrated in Supplemental Data Figure 1, *IGF1* qPCR efficiency was 96.7% with a linearity of r^2^= 0.99. The LOQ was defined as the lowest concentration that could be quantified with >80% accuracy, and set to 16 copies per reaction. LOD was found to be 4 copies.

*IGF1* primers probe were chosen to target all *IGF1* mRNA isoforms including mechano-growth factor (*MGF*) using UCSC Genome Browser (http://genome.ucsc.edu/) and Primer 3 [24].
