# Supplementary material for: Transgene Detection by Digital Droplet PCR
Source: PLoS One. 2014 Nov 6;9(11):e111781. doi: 10.1371/journal.pone.0111781 (PMC4222945; doi:10.1371/journal.pone.0111781)
Supplement: Figure S2 — PCR efficiency for undigested vs. digested IGF1 standards - Linearization of circular DNA leads to improved PCR efficiency and better detection at low copy numbers. (DOCX) [file pone.0111781.s002.docx]

**Supplemental Data Figure 2:**

**Supplemental Data Figure 2:** PCR efficiency for undigested vs. digested *IGF1* standards - Linearization of circular DNA leads to improved PCR efficiency and better detection at low copy numbers

To test for putative template effects on amplification efficiencies, two different sized plasmids containing the 462bp *IGF1* coding sequence (pAAV9-*IGF1*-5237 bp; Topo PCR 2.1-*IGF1*-4339 bp) and a PCR-generated *IGF1* standard were subjected to qPCR with or without DdeI and RsaI double-digestion. We assayed serial dilutions (ranging from 10^6^-10 copies) from 2 vectors carrying the *IGF1* coding sequence (pAAV9-*IGF1*-5237bp and TOPO-*IGF1-*4397bp) compared to linear PCR product. Improved amplification for the linear PCR standard (Ct difference of more than 3), compared to circular, supercoiled vectors was observed. Subsequently, we tested digested plasmid vs. undigested plasmid compared to linear PCR-standard and were able to amplify the digested plasmid with efficiencies close to the linear PCR-standard. Experiments were done in different independent trials in triplicates.
