# Supplementary material for: Transgene Detection by Digital Droplet PCR
Source: PLoS One. 2014 Nov 6;9(11):e111781. doi: 10.1371/journal.pone.0111781 (PMC4222945; doi:10.1371/journal.pone.0111781)
Supplement: Figure S3 — Temperature gradient for ddPCR detection of IGF1, EPO and ICS. (DOCX) [file pone.0111781.s003.docx]

**Supplemental Data Figure 3:**


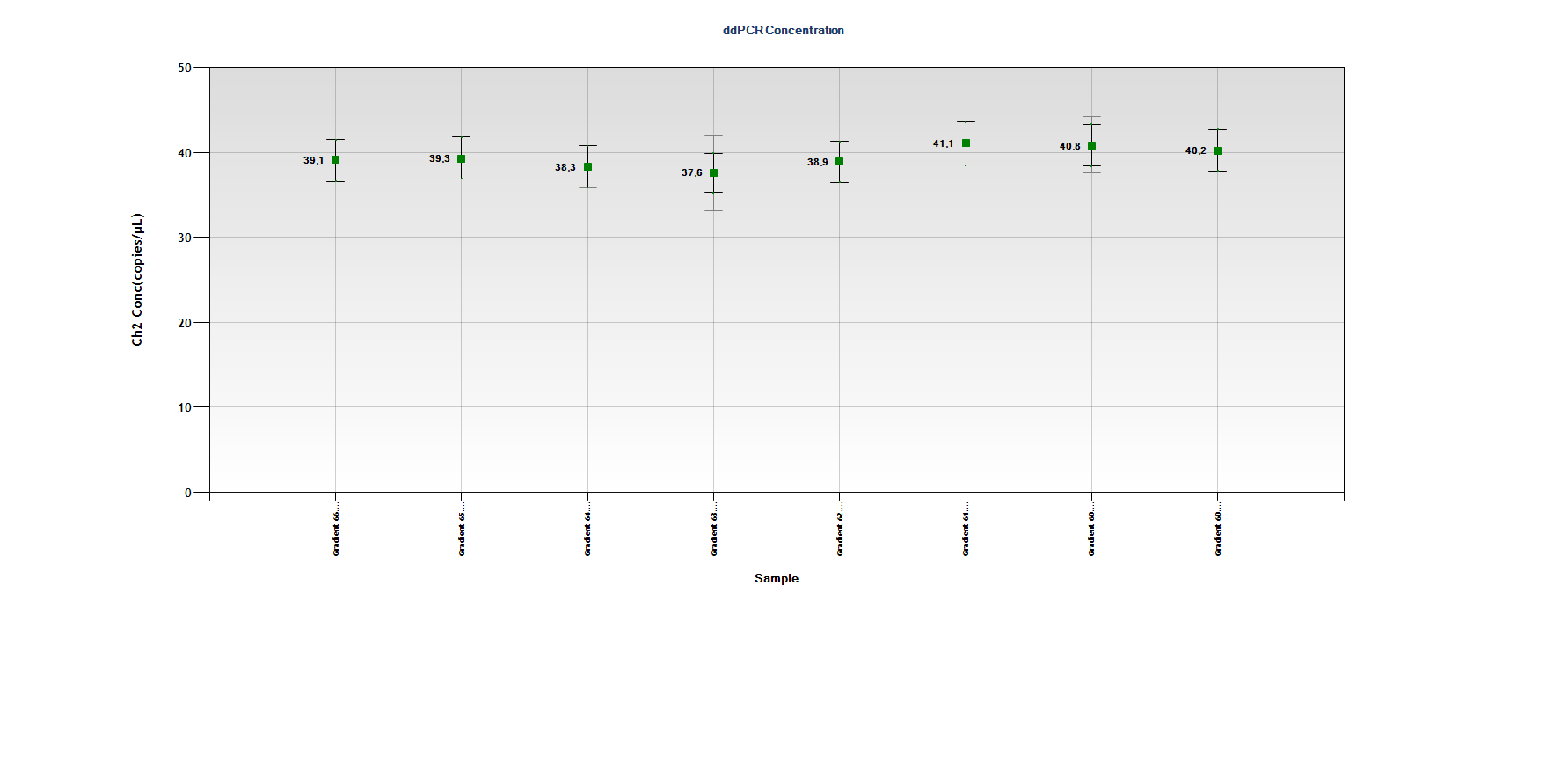

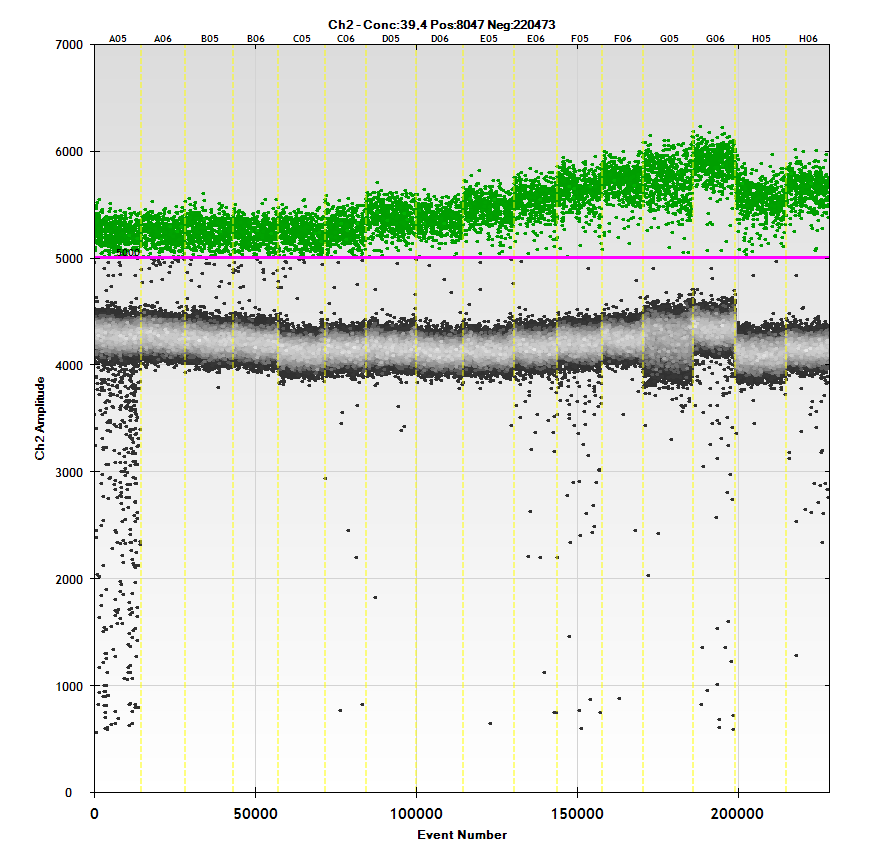

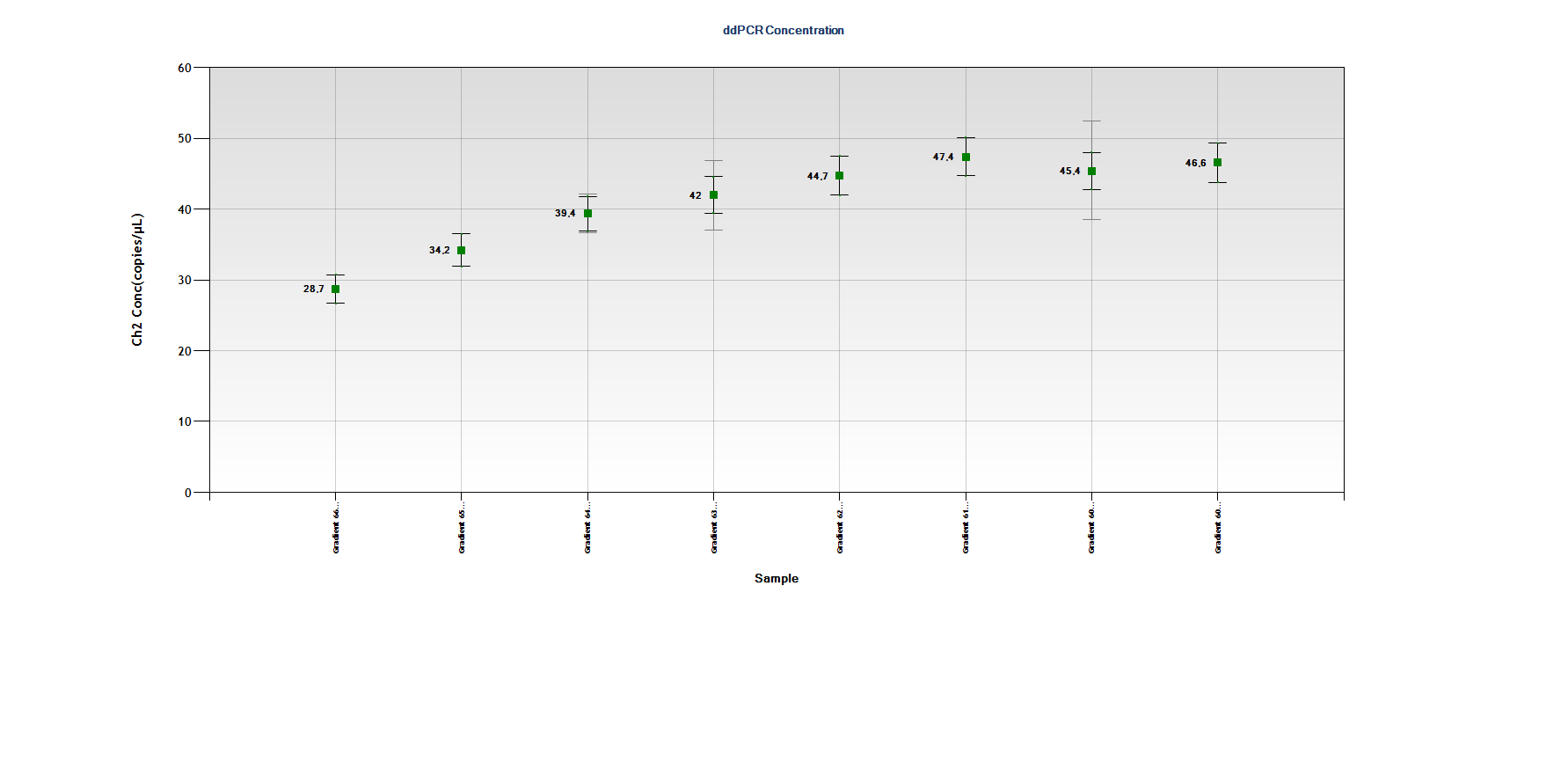

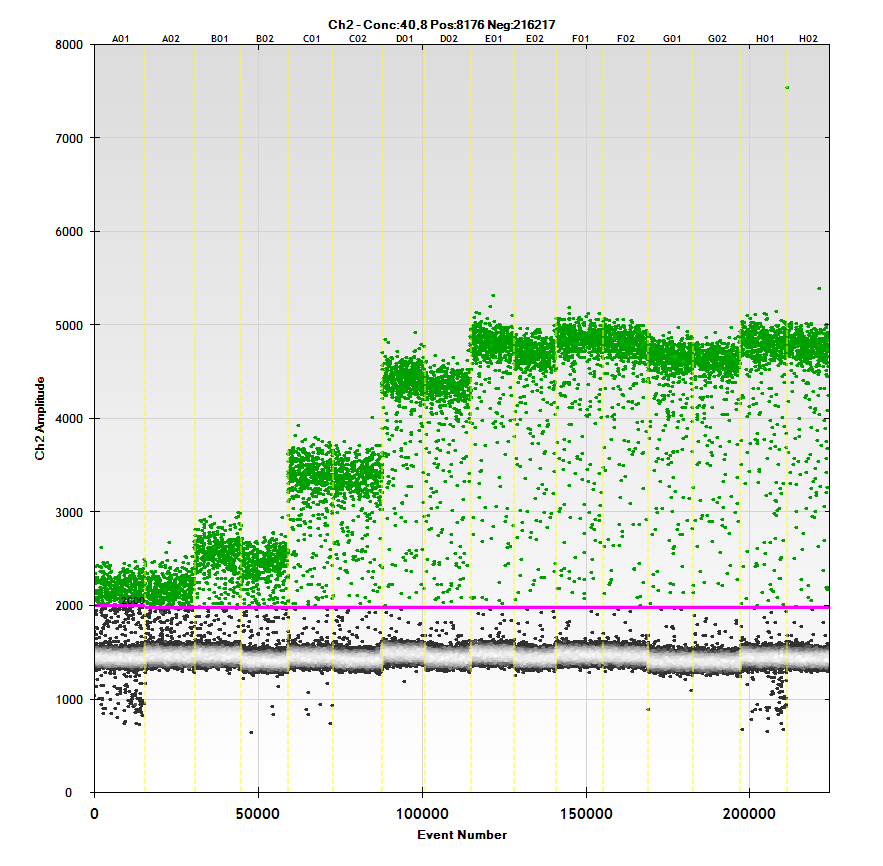

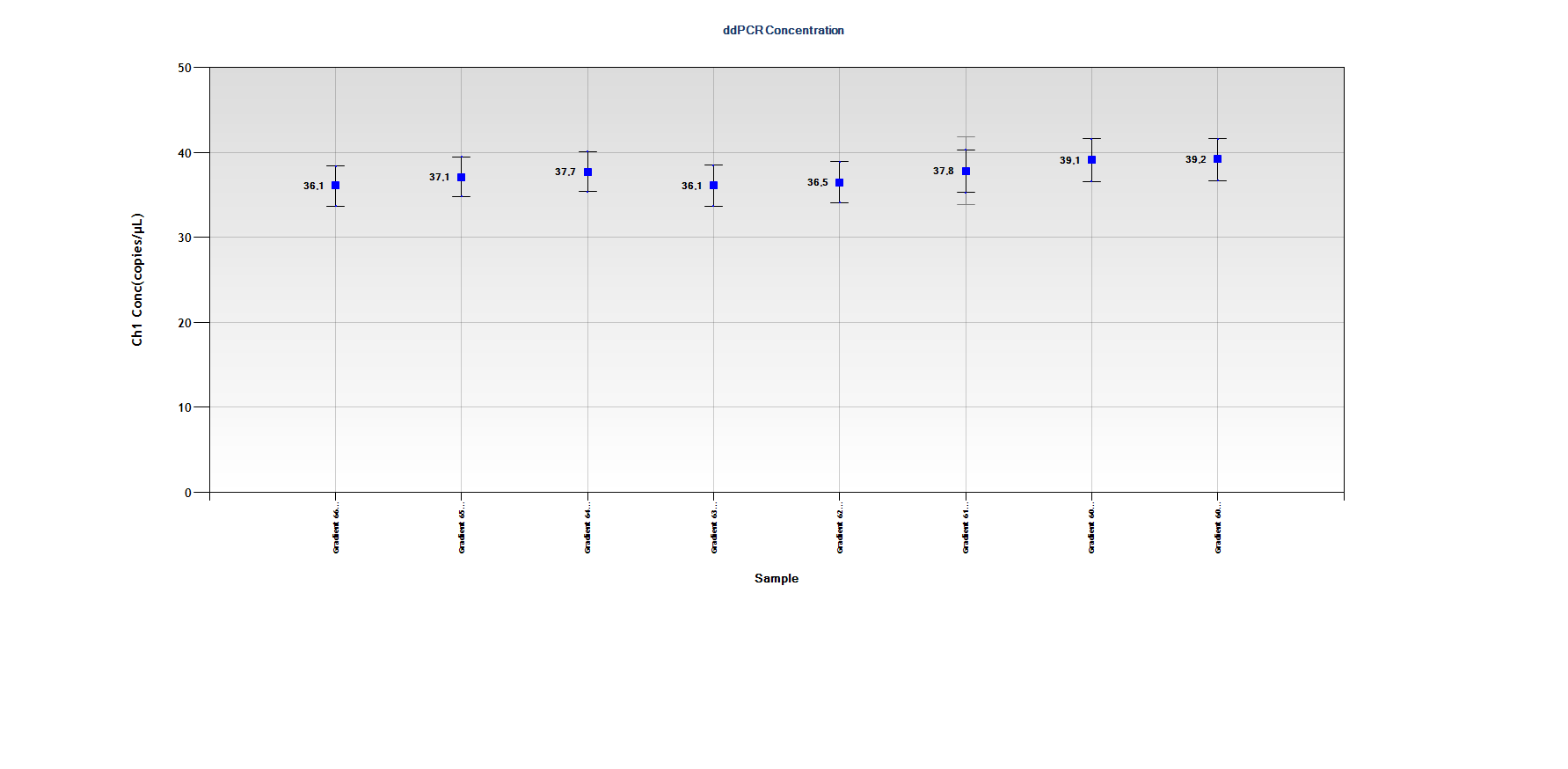

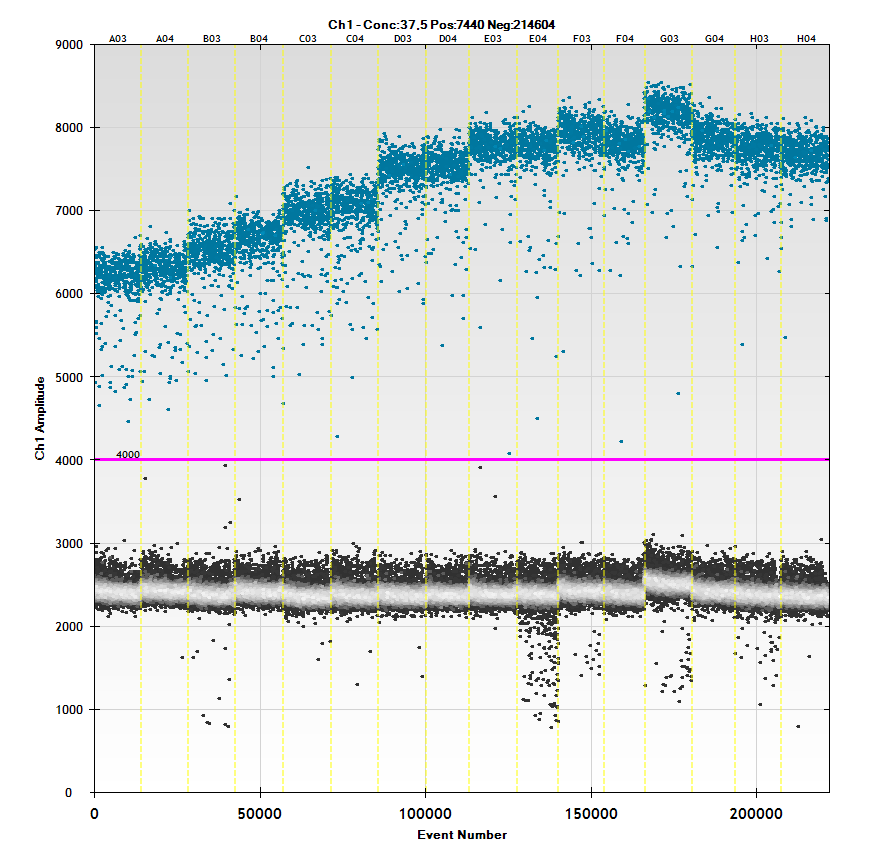


*IGF1*

*EPO*

ICS

**66°C 65.6°C 64.8°C 63.7°C 62.3°C 61.2°C 60.4°C 60°C**

**Supplemental Data Figure 3:** Temperature gradient for ddPCR detection of *IGF*1, *EPO* and ICS.

Annealing temperatures from 66°C to 60°C were tested. Displayed are concentrations/µl and amplitudes for each amplicon PCR performed in duplicates. Optimal annealing temperatures (60°C – 66°C) were assayed carrying out gradient PCR for all primers and their specific targets. As indicated in Supplemental Data Figure 3, all primers worked well between 66°C-60°C, with highest amplitude and best sensitivity at ~61°C. Subsequently, all ddPCR assays were conducted at 61°C annealing/extension temperature.
