# Supplementary material for: Transgene Detection by Digital Droplet PCR
Source: PLoS One. 2014 Nov 6;9(11):e111781. doi: 10.1371/journal.pone.0111781 (PMC4222945; doi:10.1371/journal.pone.0111781)
Supplement: Figure S4 — ddPCR efficiency under various conditions. (DOCX) [file pone.0111781.s004.docx]

**Supplemental Data Figure 4:**
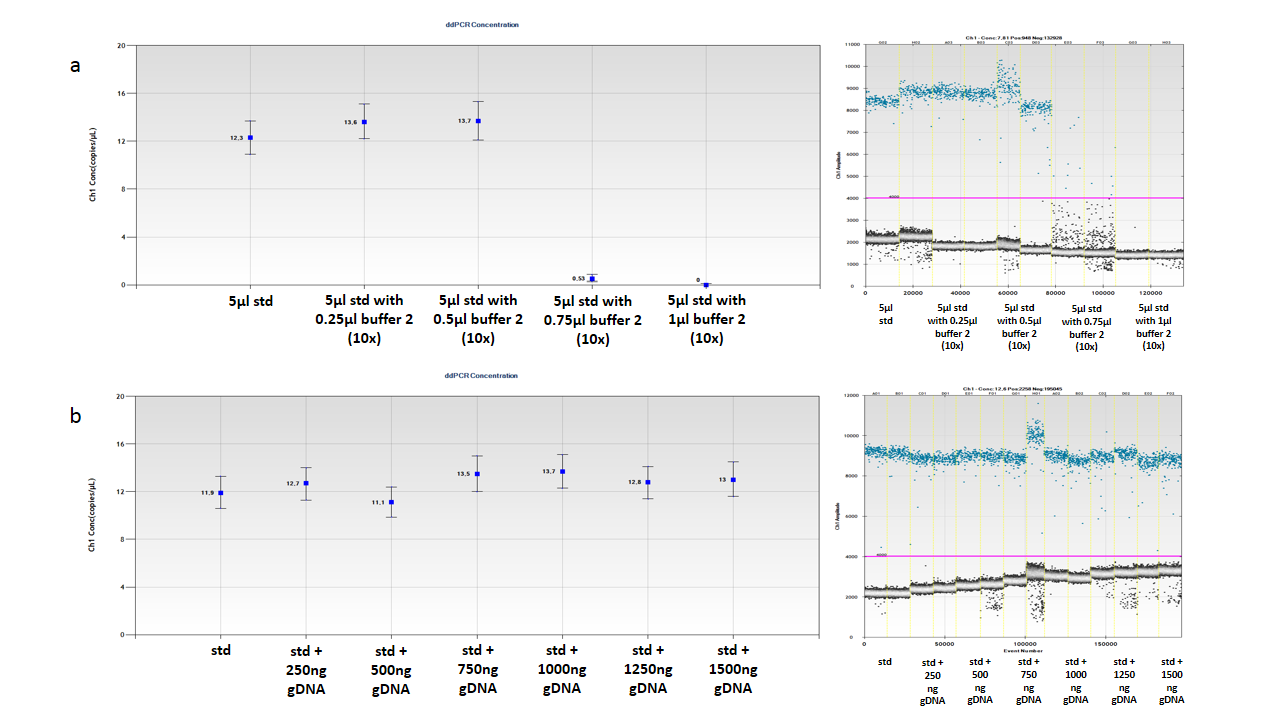


**Supplemental Data Figure 4:** ddPCR efficiency under various conditions.

1. *IGF1* ddPCR detection at increasing salt concentrations performed in duplicates

We tested for inhibitory salt effects on ddPCR efficiency by adding different amounts of buffer 2 (0 µl; 0.25 µl; 0.5 µl; 0.75 µl and 1 µl corresponding to 0 µl; 2.5 µl; 5 µl; 7.5 µl; and 10 µl of restriction solution in 20µl of ddPCR mixture) to the same amount of standard solution, We observed inhibitory effects only in those cases when more than the equivalent of 5µl DNA restriction solution were subjected into 20 µl of ddPCR mixture (Supplemental Data Figure 4a).

1. *IGF1* ddPCR detection at increasing amounts of genomic DNA performed in duplicates

Increasing amounts from 250ng up to 1500ng of genomic DNA in the background of the reaction were also assayed and did not show any inhibitory effects on ddPCR (Supplemental Data Figure 4b). Consequently, all ddPCRs were performed using a final volume of 4µl template DNA (47ng – 1500ng), which avoided potential salt and DNA inhibitory effects.
