# Supplementary material for: Transgene Detection by Digital Droplet PCR
Source: PLoS One. 2014 Nov 6;9(11):e111781. doi: 10.1371/journal.pone.0111781 (PMC4222945; doi:10.1371/journal.pone.0111781)
Supplement: Figure S5 — Qualitative assessment of ddPCR results. (DOCX) [file pone.0111781.s005.docx]

**Supplemental Data Figure 5:**


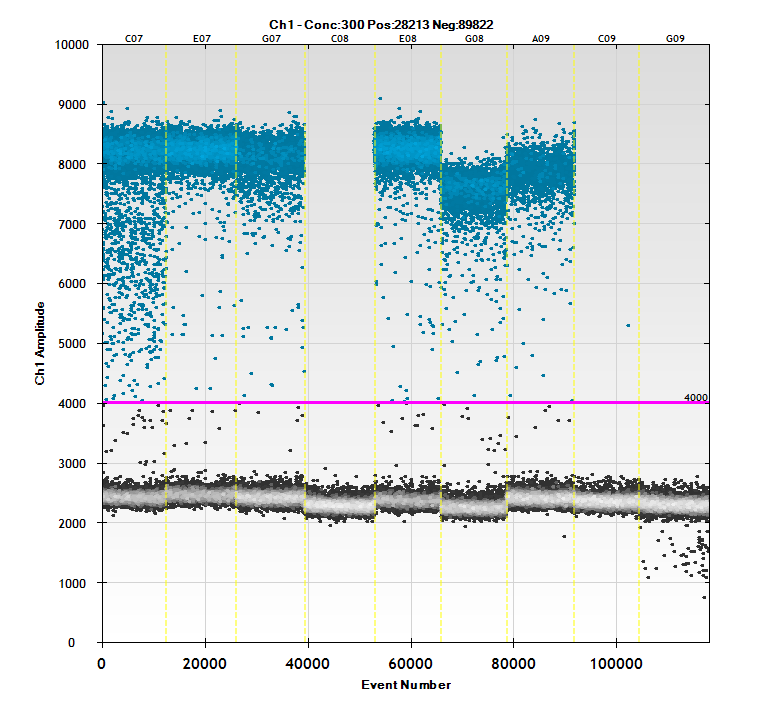


**A B C D E F G H H_2_O**


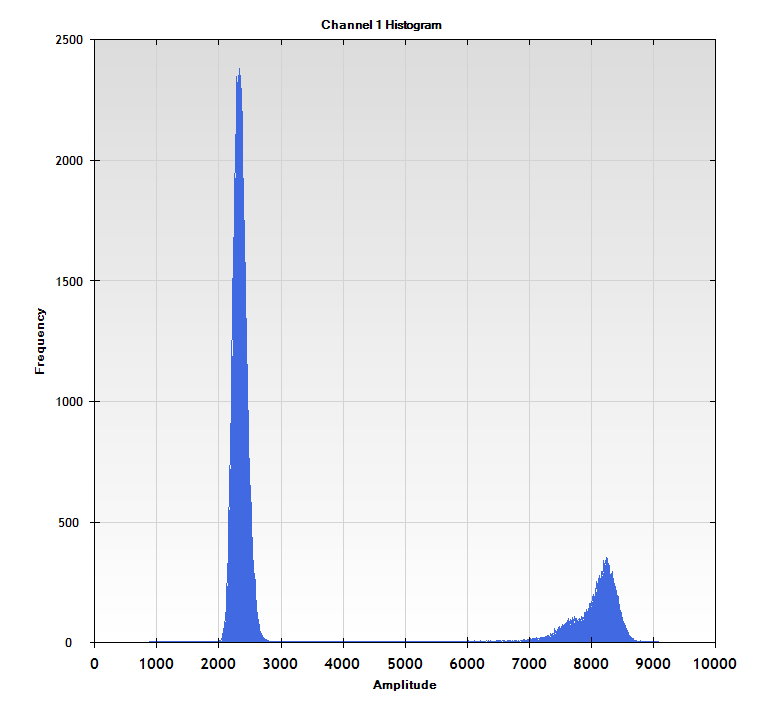


**Supplemental Data Figure 5:** Qualitative assessment of ddPCR results

Doing digital PCR, false positive events can be clearly discriminated from true positives by manual re-adjustment of the threshold to a value that defined the lower limit of the positive control, a best practice for ddPCR, as also discussed earlier elsewhere [26]. Furthermore, graphical illustration allows for qualitative assessment of ddPCR results, whereas nested PCR, for instance, only allows for a Yes/No evaluation
