# Supplementary material for: Transgene Detection by Digital Droplet PCR
Source: PLoS One. 2014 Nov 6;9(11):e111781. doi: 10.1371/journal.pone.0111781 (PMC4222945; doi:10.1371/journal.pone.0111781)
Supplement: Figure S6 — Concentration, purity and integrity of DNA comparing three different DNA extraction procedures. (DOCX) [file pone.0111781.s006.docx]

**Supplemental Data Figure 6:**

**Supplemental Data Figure 6:** Concentration, purity and integrity of DNA comparing three different DNA extraction procedures.

Displayed are concentrations (ng/µl) and purity (260/280 and 260/230) as revealed by Nanodrop for 5 subjects performed in duplicates. Integrity and yield of the same volumes of DNA analysed by agarose-gel are displayed on the right.
