# Supplementary material for: Transgene Detection by Digital Droplet PCR
Source: PLoS One. 2014 Nov 6;9(11):e111781. doi: 10.1371/journal.pone.0111781 (PMC4222945; doi:10.1371/journal.pone.0111781)
Supplement: Table S1 — IGF1 transgene copy numbers as detected by ddPCR for 6 AAV9-IGF1 transduced mice and 2 controls at 5 different days. (DOCX) [file pone.0111781.s007.docx]

**SUPPLEMENTAL DATA TABLE 1**

*IGF1* transgene copy numbers as detected by ddPCR for 6 AAV9-*IGF1* transduced mice and 2 controls at 5 different days.


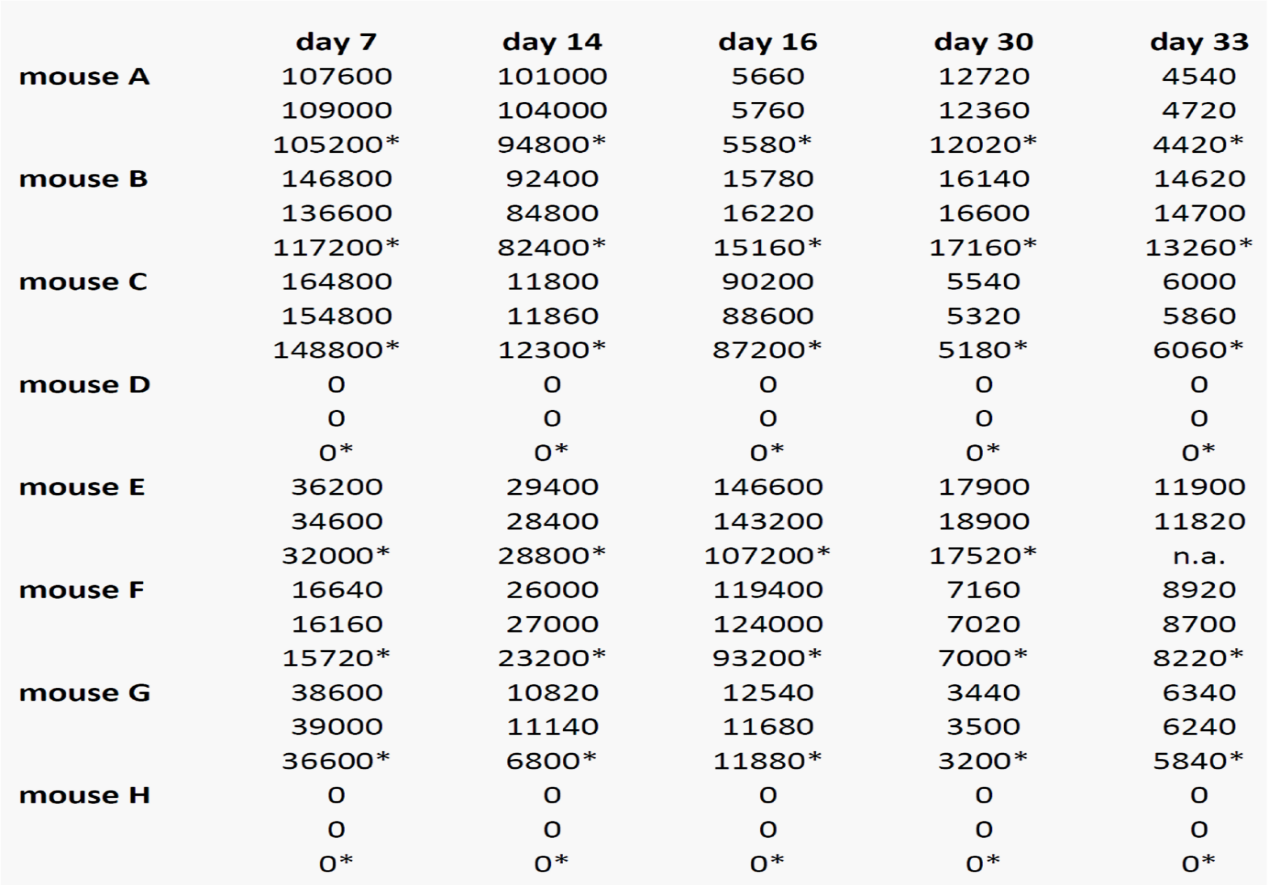


Data were achieved by two independent experiments. The first ddPCR was done in duplicates - the second after one additional freezing and thawing cycle as singletons (*).
